# Molecular determinants of Ras-mTORC2 signaling

**DOI:** 10.1016/j.jbc.2024.107423

**Published:** 2024-05-28

**Authors:** Stephen F. Smith, A.F.M. Tariqul Islam, Shoxruxxon Alimukhamedov, Ethan T. Weiss, Pascale G. Charest

**Affiliations:** 1 Department of Chemistry and Biochemistry, University of Arizona, Tucson, Arizona, USA; 2 Department of Molecular and Cellular Biology, University of Arizona, Tucson, Arizona, USA; 3 University of Arizona Cancer Center, Tucson, Arizona, USA

**Keywords:** RasC, mTOR, mechanistic Target of Rapamycin complex 2, *Dictyostelium*, switch I, hypervariable region, HVR, allosteric domain

## Abstract

Recent research has identified the mechanistic Target of Rapamycin Complex 2 (mTORC2) as a conserved direct effector of Ras proteins. While previous studies suggested the involvement of the Switch I (SWI) effector domain of Ras in binding mTORC2 components, the regulation of the Ras-mTORC2 pathway is not entirely understood. In *Dictyostelium*, mTORC2 is selectively activated by the Ras protein RasC, and the RasC-mTORC2 pathway then mediates chemotaxis to cAMP and cellular aggregation by regulating the actin cytoskeleton and promoting cAMP signal relay. Here, we investigated the role of specific residues in RasC's SWI, C-terminal allosteric domain, and hypervariable region (HVR) related to mTORC2 activation. Interestingly, our results suggest that RasC SWI residue A31, which was previously implicated in RasC-mediated aggregation, regulates RasC’s specific activation by the Aimless RasGEF. On the other hand, our investigation identified a crucial role for RasC SWI residue T36, with secondary contributions from E38 and allosteric domain residues. Finally, we found that conserved basic residues and the adjacent prenylation site in the HVR, which are crucial for RasC’s membrane localization, are essential for RasC-mTORC2 pathway activation by allowing for both RasC’s own cAMP-induced activation and its subsequent activation of mTORC2. Therefore, our findings revealed new determinants of RasC-mTORC2 pathway specificity in *Dictyostelium*, contributing to a deeper understanding of Ras signaling regulation in eukaryotic cells.

Ras proteins are monomeric GTPases and notorious oncoproteins involved in the transduction of various extracellular signals and best characterized for their roles in promoting cell survival, cell growth, and proliferation downstream from growth factor receptors (1). Other cellular roles of Ras include the control of differentiation, apoptosis, vesicular trafficking, cell junction, and adhesion, as well as cell motility and migration (1, 2). Ras proteins are often described as molecular switches that cycle between inactive GDP-bound and active GTP-bound forms, which are regulated by guanine nucleotide exchange factors (GEFs) and GTPase activating proteins (GAPs) (3, 4, 5, 6). Typically, a stimulus leads to the activation of a RasGEF that then interacts with inactive GDP-bound Ras, which destabilizes the interaction of Ras with the nucleotide leading it to dissociate and be replaced by abundant cellular GTP. GTP binding then causes a change in conformation in two highly conserved regions of Ras, switch I (SWI) and switch II (SWII), exposing interface residues involved in canonical effector binding (7). Ras-GTP then activates many different signaling pathways, including those mediated by the well-described Ras effectors Raf1, phosphoinositide 3-kinase (PI3K), and RalGDS, that contain defined Ras binding domains (RBDs) (2). At the core of the interaction of Ras with these domains are conserved residues in SWI, including T35, E37, D38, and Y40, termed the effector binding domain (8, 9, 10, 11, 12, 13). However, increasing evidence indicates that Ras’s cellular functions also involve interacting with non-canonical effectors that lack typical RBDs (14). Moreover, previous studies suggest that Ras’s interaction with non-canonical effectors is likely to involve its allosteric domain, with a possible role of residues R135, R161, and R164 (proposed to be important for Ras clustering and function), and the short C-terminal hypervariable region (HVR) that is only 10% conserved between Ras proteins (14).

We previously reported the discovery of the mechanistic Target of Rapamycin Complex 2 (mTORC2) as a non-canonical effector of Ras in the experimental model *Dictyostelium discoideum* (15), which was later shown to be conserved in human cells (4, 5). mTORC2 is one of two signaling complexes formed by the serine/threonine kinase mTOR and is involved in the regulation of metabolism, cell survival, and cell migration (16, 17). mTORC2’s functional specificity compared to mTORC1 is determined by its unique components, including the proteins Rictor and SIN1 (16). Although SIN1 contains a Ras binding-related domain that was shown to bind human Ras proteins and the *Dictyostelium* Ras-related protein Rap1 *in vitro* (4, 5, 6, 15, 18), we and others also found that Ras can directly bind mTOR *in vitro*, suggesting that the kinase is a potential non-canonical Ras effector (4, 15). In addition, evidence indicates that residues T35 and Y40 in the SWI domain of human Ras are important for its interaction with mTOR *in vitro* and its activation of mTORC2 in cells (4).

In *Dictyostelium*, two closely related homologs of human Ras proteins, RasC and RasG, are key mediators of the response to chemoattractants and promote directed cell migration (19, 20, 21, 22). Interestingly, only RasC specifically activates mTORC2 (15, 23, 24, 25). The RasC-mTORC2 pathway controls F-actin dynamics and is necessary for cAMP production and signal relay during chemotaxis, which mediates the aggregation of cells during development (15, 21, 25). On the other hand, RasG binds and activates PI3K and, thereby, the downstream PI(3,4,5)P_3_-dependent signaling pathways that promote F-actin polymerization and chemotaxis (21, 26, 27, 28). Both RasG-PI3K and RasC-mTORC2 pathways promote the activation of AKT kinases, which are crucial to the signal relay and chemotactic response (23, 24, 25, 27, 29, 30, 31, 32). *Dictyostelium* AKT/Protein Kinase B (PKB), like mammalian AKT, is cytosolic and recruited to the plasma membrane through its Plextrin Homology (PH) domain binding to PI(3,4,5)P_3_ and, thereby, is regulated by PI3K. By contrast, PKBR1 is permanently localized to the plasma membrane through an N-terminal myristoyl anchor and is not regulated by PI3K (23, 24, 29, 30). However, both PKB and PKBR1 are regulated by mTORC2 through its phosphorylation of the kinases at their hydrophobic motif (HM), specifically T473 in PKB and T470 in PKBR1 (23, 24, 25, 32, 33).

How RasC selectively activates mTORC2 is not understood, but an earlier study revealed that RasC’s amino acid residue A31 in SWI (aspartate in RasG) as well as its C-terminal half are important determinants of RasC’s unique role in *Dictyostelium* aggregation (34). Moreover, residues T26, Q28, and N39 in SWI, which are also unique to RasC compared to RasG, were determined to have no impact on aggregation (34). Here, to gain additional insight into the molecular determinants of RasC’s activation of mTORC2, we further examined the role of A31 as well as of other SWI residues involved in canonical Ras effector interactions and investigated the role of specific residues and motifs in the C-terminal allosteric domain and HVR.

## Results and discussion

### Residues in SWI and C-terminal domain determine RasC function in aggregation

**Figure 1:**
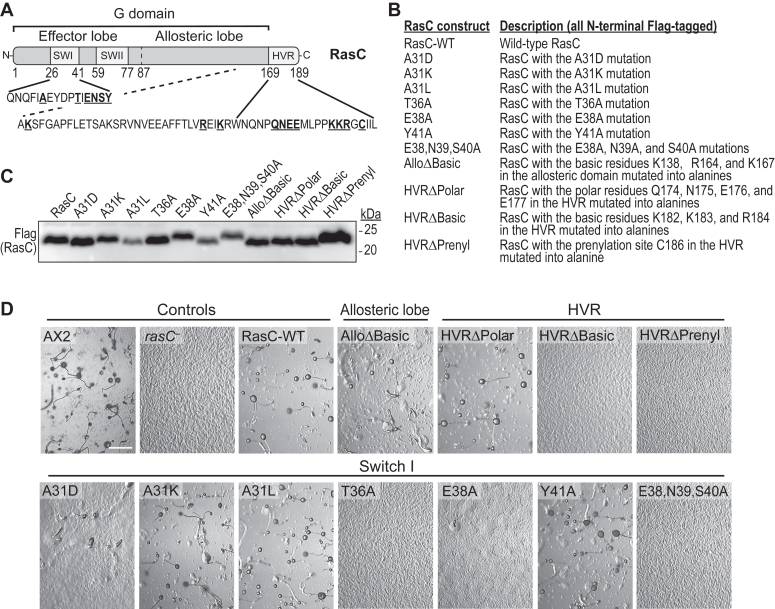
**RasC residues and domains studied and the effect of their mutation on starvation-induced aggregation.***A*, RasC domain organization showing the location of the residues mutated. *B*, RasC mutants used in the study. *C*, the expression of each Flag-tagged RasC construct was detected by Flag immunoblot. *D*, the indicated *Dictyostelium* strains were plated on non-nutrient agar, and their development was monitored for 48 h. Pictures shown were taken 48 h after onset of starvation and are representative of at least three independent experiments. The location of specific mutations within defined regions of RasC is indicated. Scale bar, 10 μm.

To address the role of specific residues in the SWI, allosteric domain, and HVR of RasC on its activation of mTORC2, we first replaced them with residues of different chemistries (Fig. 1, *A* and *B*). In SWI, we mutated residue A31 to aspartate (negative charge and residue at this position in RasG; A31D), lysine (positive charge; A31K), and leucine (bulkier hydrophobic; A31L); and we mutated T36, E38, N39, S40, and Y41 to alanine, separately or in combination (T36A, E38A, Y41A, E38,N39,S40A). These residues correspond to the highly conserved SWI residues T35, E37, D38, S39, and Y40 in human Ras proteins that are part of the canonical effector binding domain (Fig. S1). In the allosteric domain, we mutated the basic residues K138, R164, and K167 to alanine (corresponding to residues R135, R161, and R164 in human Ras; AlloΔBasic). These residues were previously suggested to promote human Ras dimerization and/or clustering at the membrane and to mediate interactions with non-canonical effectors (14, 35, 36, 37, 38, 39). Of note, while we were conducting this study, another group reported that residues in RasC’s allosteric domain, including R164, mediate its interaction with the Rho GTPase RacE and that this is important for binding and activating mTORC2 (40, 41). Finally, we mutated HVR polar residues Q174, N175, E176, and E177 to alanine (HVRΔPolar), the adjacent basic residues K182, K183, and R184 to alanine (HVRΔBasic), and the conserved prenylation site C186 to alanine (HVRΔPrenyl). The polar residues in RasC’s HVR are absent in RasG but conserved in human Ras proteins (Fig. S1), and the three basic residues correspond to residues in another small GTPase, Rac1, found to be implicated in its interaction with mTOR (42).

Each RasC mutant was tagged with Flag at the N-terminus and expressed in *rasC* null cells, and we used wild-type Flag-RasC-expressing *rasC* null cells (RasC-WT) as reference control in all experiments. All mutants displayed considerable expression, with most levels comparable to wild-type RasC, except for A31L and Y41A which were often expressing at lower levels and HVRΔPrenyl that displayed consistently higher expression levels (Fig. 1*C*). Since RasC is essential for *Dictyostelium*’s development and that *rasC* null cells fail to aggregate when starved (21, 22), we first evaluated the ability of the RasC mutants to rescue this aggregation defect. As expected, we observed that *rasC* null cells fail to aggregate and that the exogenous expression of RasC rescues the cells’ aggregation and development (Fig. 1*D*). Interestingly, the distinct A31 mutations produced different effects: A31D displayed severe aggregation defects whereas A31K and A31L fully rescued the development of *rasC* null cells. As for the mutations in SWI, T36A, E38A, and triple mutant E38, N39, S40A did not promote aggregation whereas Y41A fully rescued *rasC* null cells’ development. The allosteric domain mutant, AlloΔBasic, partially rescued the development, displaying some aggregation and formation of multicellular structures, but no mature fruiting bodies. Finally, the HVRΔPolar fully rescued development whereas HVRΔBasic and HVRΔPrenyl completely failed to promote aggregation and development. These observations indicate that residue A31 plays an important role in RasC’s cellular function, as previously reported, and that the presence of aspartate in this position (as found in RasG) is particularly disrupting. Our observations further indicate that SWI residues T36 and E38 as well as the basic residues and prenylation site in RasC’s HVR are crucial for RasC’s role in mediating aggregation, whereas residues K138, R164, and K167 in the allosteric domain (AlloΔBasic mutant) are less important.

### SWI and C-terminal domain RasC residues regulate mTORC2 pathway activation in cells

**Figure 2:**
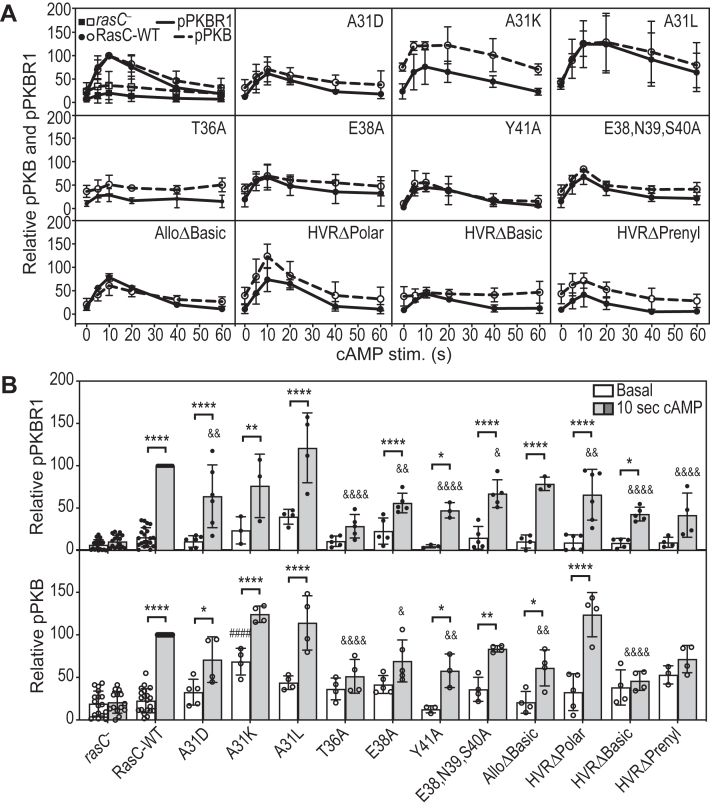
**Impact of the RasC mutations on mTORC2-mediated pPKB and pPKBR1 in cells.** Activation of the RasC-mediated mTORC2-PKB/PKBR1 pathway in response to cAMP stimulation of cells expressing the indicated Flag-tagged RasC proteins was assessed by evaluating the phosphorylation of PKB at T435 (pPKB) and PKBR1 at T470 (pPKBR1) by immunoblot (Fig. S2). Immunoblots were quantified by densitometry and expressed relative to the peak activity of wild-type RasC (10 s) as described in the Experimental procedures section. Data on graphs represent measured pPKB and pPKBR1 ± S.D of at least three independent experiments for each strain. *A*, kinetics of cAMP-induced pPKB and pPKBR1. *B*, comparison of pPKB and pPKBR1 levels before and 10 s after cAMP stimulation. Data were analyzed using a one-way ANOVA test followed by Bonferroni correction. Adjusted *p* values for the difference between basal and cAMP stimulated condition for each strain: ∗*p* < 0.05; ∗∗*p* < 0.005; ∗∗∗*p* < 0.0005; ∗∗∗∗*p* < 0.0001. Adjusted *p* values for the difference between the basal levels of cells expressing the RasC mutants *versus* wild-type RasC: ####*p* < 0.0001. Adjusted *p* values for the difference between the cAMP stimulated levels of cells expressing the RasC mutants *versus* wild-type RasC: &*p* < 0.05; &&*p* < 0.005; &&&&*p* < 0.0001.

We then assessed the ability of the RasC mutants to promote activation of the mTORC2 pathway in cells in response to cAMP stimulation. For this, we developed the cells for 5.5 h using external cAMP pulses, which allows cells unable to produce cAMP such as the *rasC*- cells to induce the developmental program (expression of developmental genes) independently of chemotaxis and aggregation and generate cells highly responsive to the cAMP chemoattractant (21, 22). We then monitored the cAMP-induced, mTORC2-mediated phosphorylation of its substrates PKB and PKBR1 (21, 22, 23, 25, 43) (Figs. 2 and S2). All three A31 mutants displayed the ability to promote pPKB and pPKBR1 stimulation, but A31D showed lower activity levels (Fig. 2), potentially explaining its developmental defect compared to A31K and A31L (Fig. 1*C*). Of the other SWI mutations under study, we did not detect any significant pPKB and pPKBR1 stimulated in T36A expressing cells, whereas E38A, Y41A, and E38,N39,S40A displayed similar pPKB and pPKBR1 stimulation profiles, which are reduced compared to wild-type RasC, although some of the differences do not show statistical significance. As for the mutations in the C-terminal portion of RasC, AlloΔBasic and HVRΔPolar showed significant stimulation of pPKB and pPKBR1, although AlloΔBasic showed reduced pPKB and HVRΔPolar showed lower pPKBR1 (Fig. 2). On the other hand, HVRΔBasic and HVRΔPrenyl display little pPKB and pPKBR1 responses, with only significant stimulation of pPKBR1 by HVRΔBasic detected (Fig. 2*B*).

Therefore, in general, we found a connection between several of the mutants’ ability to promote mTORC2 activation and their aggregation defects: A31K, A31L, and HVRΔPolar are able to promote strong mTORC2 pathway activation and they are able to aggregate; A31D, AlloΔBasic, and Y41A display reduced mTORC2 pathway activity and partially rescue aggregation and development; Y41A and T36A, HVRΔBasic, and HVRΔPrenyl have strongly impaired or completely lack mTORC2 pathway activation and do not aggregate. On the other hand, the aggregation and mTORC2 pathway activity phenotypes do not correlate for the E38A and E38,N39,S40A mutants. E38A and E38,N39,S40A activate the mTORC2 pathway at levels similar to those of AlloΔBasic; however, these mutants completely fail to rescue the aggregation of *rasC* null cells (Figs. 1*D* and 2*B*). Thus, these observations suggest that E38A and E38,N39,S40A possibly affect an mTORC2-independent role of RasC in aggregation.

### The HVR and allosteric domain promote the association of RasC with membranes

**Figure 3:**
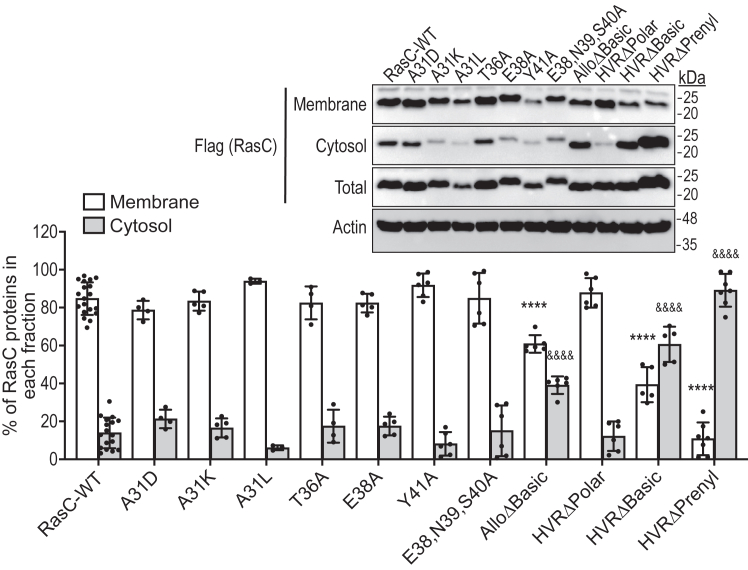
**The membrane association profiles of the different RasC mutants.** The proportion of RasC associated with membranes in cells expressing the different Flag-tagged Ras proteins was assessed using membrane/cytosol separation followed by immunoblotting and quantification by densitometry as described in the Experimental procedures section. RasC proteins were detected in the membrane and cytosolic fractions using Flag immunoblot. The immunoblot of Flag-RasC proteins and actin in total cell lysates were used as expression and loading controls, respectively. The proportion of RasC proteins in the membrane and cytosolic fractions were quantified and plotted as % of RasC proteins detected in each fraction from at least three independent experiments ± S.D. Differences between levels of RasC mutant proteins detected in each fraction were statistically compared to the levels of wild-type RasC in the corresponding fractions using a one-way ANOVA test followed by Bonferroni correction. Adjusted *p* values for the membrane levels: ∗∗∗∗*p* < 0.0001. Adjusted *p* values for the cytosol levels: &&&&*p* < 0.0001. *Inset*, immunoblots representative of three independent experiments. Note: the total Flag-RasC immunoblot is the same as that used in Figure 1*C*.

RasC is activated by the Aimless/RasGEFA-containing Sca1 complex, which is transiently recruited to the plasma membrane upon chemoattractant stimulation (25). In addition, evidence suggests that mTORC2 is activated at the plasma membrane in response to cAMP stimulation (23). Therefore, since the localization of RasC to the plasma membrane could affect its activation by the ScaI complex and its activation of mTORC2, we evaluated how the mutations in SWI and C-terminal domain affect RasC’s membrane localization. For this, we isolated the cytosolic and membrane fractions of cells expressing each of the RasC mutants and determined their relative amount in each fraction by immunoblot followed by densitometric analyses. As expected, we observed the majority (∼85%) of wild-type RasC associated with membranes (Fig. 3). Like wild-type RasC, we found that all SWI mutants as well as the HVRΔPolar mutant are primarily associated with membranes. On the other hand, we observed that the AlloΔBasic (∼60%), the HVRΔBasic (∼40%), and HVRΔPrenyl (∼10%) mutants display significantly reduced association with the membrane fraction (Fig. 3*B*). The finding that mutation of the prenylation site and adjacent basic residues in RasC’s HVR result in reduced membrane association was not surprising as these have been extensively studied and showed to promote membrane anchoring of orthologous human Ras proteins (44, 45, 46). However, our results also suggest a secondary role for the allosteric domain, which may be due to RasC’s interaction with Rho GTPases, which was found to involve this region of RasC (41). Therefore, for these three mutants, there seem to be a correlation between their membrane localization and their ability to promote mTORC2 pathway activation (Fig. 2*B*).

### SWI residue A31 and membrane localization affect cAMP-induced RasC activation

**Figure 4:**
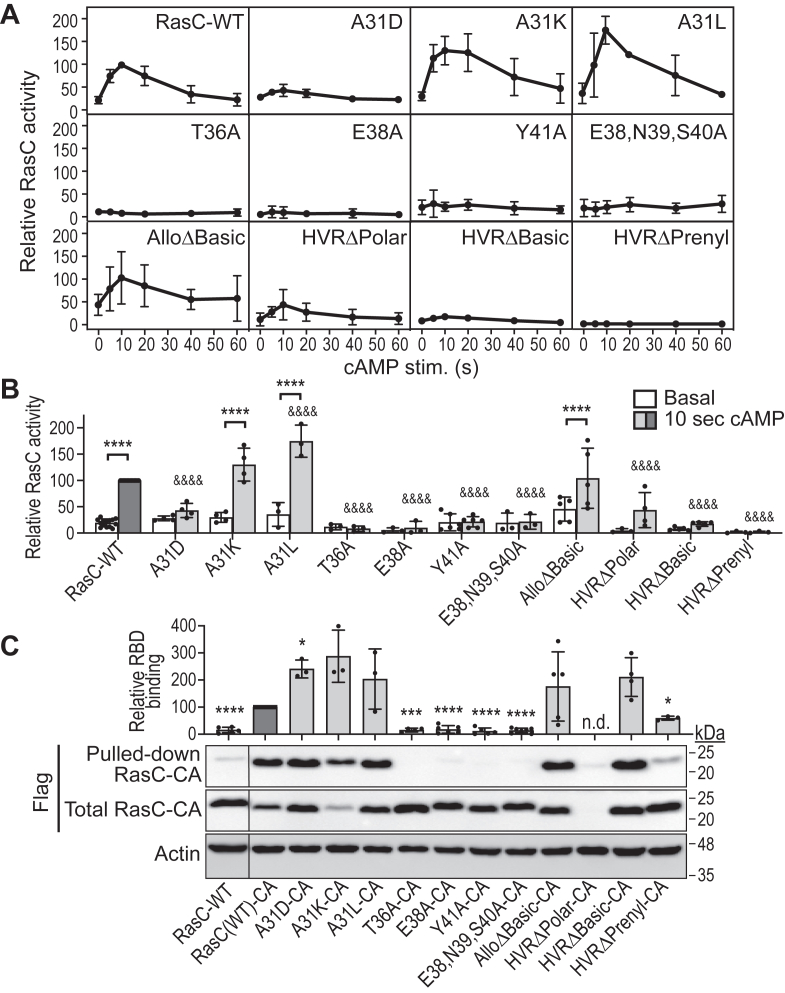
**Differential effects of the RasC mutations on their own cAMP-induced activation and canonical effector binding.***A* and *B*, the activity of the RasC proteins before and upon cAMP stimulation was measured using a pull-down assay with Byr2(RBD) followed by Flag immunoblot (Fig. S3). Immunoblots were quantified by densitometry and RasC activity was expressed relative to the peak activity of wild-type RasC (10 s) as described in the Experimental procedures section. Data represent relative RasC activity measured from at least three independent experiments for each strain ± S.D. *A*, kinetics of measured RasC activity in response to cAMP stimulation. *B*, comparison of RasC activity levels before and after 10 s cAMP stimulation. Data were analyzed using a one-way ANOVA test followed by Bonferroni correction. Adjusted *p* values for the difference between basal and cAMP-stimulated levels for each strain: ∗∗∗∗*p* < 0.0001. Adjusted *p* values for the difference between the RasC activity levels 10 s after cAMP stimulation in cells expressing the RasC mutants *versus* wild-type RasC: &&&&*p* < 0.0001. *C*, the binding of wild-type RasC and constitutively active (CA; Q62L) forms of the RasC proteins to Byr2(RBD) was assessed in a pull-down assay and revealed by Flag immunoblot. Actin immunoblot was used as loading control. Immunoblots were quantified by densitometry and Byr2(RBD) binding was expressed relative to the binding of wild-type RasC-CA as described in the Experimental procedures section. Immunoblots shown are representative of at least three independent experiments. Data on graph represent relative Byr2(RBD) binding from at least three independent experiments for each strain ± S.D. Data were analyzed using unpaired two-tailed t-tests. ∗*p* < 0.05; ∗∗∗*p* < 0.0005; ∗∗∗∗*p* < 0.0001. *n.d.*, not determined.

To verify the activity of the RasC mutants, we then assessed their basal and cAMP-induced activation using a pull-down assay with the RBD of the yeast canonical Ras effector Byr2, which was previously shown to efficiently bind active, GTP-bound RasC (Figs. 4, *A* and *B*, S3) (47). In parallel, we evaluated the ability of the mutants to bind Byr2(RBD) independently of their GEF-mediated activation by adding the constitutively active (CA) mutation Q62L (Fig. 4*C*) (24). We observed basal activity levels of wild-type RasC in 5.5h-developed cells (∼20% of maximal stimulated activity) and its transient, cAMP-stimulated activation in cells with a peak at 10 s (Fig. 4, *A* and *B*). We also detected some Byr2(RBD) binding of wild-type RasC from non-stimulated cells (∼15% of RasC-CA; indicative of low basal activity levels) compared to that of the RasC-CA mutant (Fig. 4*C*).

Interestingly, whereas all three A31 mutants efficiently bound Byr2(RBD) (Fig. 4*C*), we detected negligible cAMP-induced activation of the A31D mutant compared to strong activations of A31K and A31L, with the latter showing levels significantly higher than wild-type RasC (Fig. 4, *A* and *B*). These observations suggest that the A31D mutation affects the cAMP-induced activation of RasC, whereas the A31L mutation (bulkier hydrophobic residue substitution) increases its activation. A31K, which is also a bulkier residue substitution with the addition of a positive charge, shows a tendency for increased RasC activity as well, but this was found to be non-statistically significant compared to wild-type RasC activity levels (Fig. 4*B*). On the other hand, we observed that mutations in the canonical effector binding domain, T36A, E38A, Y41A, and E38,N39,S40A, all strongly impair Byr2(RBD) binding (Fig. 4*C*). Therefore, the absence of measured activity for these mutants in the pull-down assay is inconclusive (Fig. 4, *A* and *B*). However, our finding that cells expressing E38A, Y41A, and E38,N39,S40A all promote mTORC2 pathway activation in cells, albeit reduced, does suggest that these three mutants are activated in response to cAMP stimulation (Fig. 2).

AlloΔBasic showed efficient binding to Byr2(RBD) and significant cAMP-stimulated activation that is comparable to wild-type RasC, indicating that this mutant’s activity is intact (Fig. 4). By contrast, the three HVR mutants displayed either significantly reduced or no detected activity (Fig. 4, *A* and *B*). HVRΔPolar showed some stimulated activity with ∼45% of that of wild-type RasC, although this stimulation was not found to be statistically significant. However, we were unable to quantitatively analyze this mutant’s interaction with Byr2(RBD) due to extremely low expression levels of HVRΔPolar with the added CA alteration (Fig. 4*C*). Nonetheless, the fact that cells expressing HVRΔPolar can promote activation of the mTORC2 pathway in cells suggests that this mutant is activated at least to some extent in response to cAMP stimulation (Fig. 2). On the other hand, HVRΔBasic displays negligeable cAMP-stimulated activity although HVRΔBasic-CA shows strong Byr2 binding, whereas HVRΔPrenyl does not show any activation and HVRΔPrenyl-CA displays reduced binding to Byr2 (∼60%) compared to wild-type RasC-CA (Fig. 4). Since both HVRΔBasic and HVRΔPrenyl mutants’ association with membranes is impaired, these observations suggest that reduced association with membranes may affect the ability of these RasC mutants to be efficiently activated in response to cAMP. These observations also suggest that lack of RasC prenylation also affects its interaction with Byr2. It is intriguing that we measured minimal cAMP-induced activation of A31D and HVRΔBasic mutants (not statistically significant) while we observed that *rasC* null cells expressing these mutants show cAMP-induced mTORC2 activation (Fig. 2*B*). Perhaps these observations indicate that the minimal RasC activation in these mutants is enough to produce a measurable mTORC2-dependent PKB and PKBR1 phosphorylation, which could be due to amplification.

To summarize these experiments, the results showed that RasC activation is unaffected in the AlloΔBasic mutant, is increased in A31K and A31L, is decreased in HVRΔPolar, is minimal in A31D and HVRΔBasic, and is absent in HVRΔPrenyl; and although the activity of E38A, Y41A and E36,N39,S40A could not be measured, the fact that these mutants promote mTORC2 pathway activation upon cAMP stimulation in cells suggest that these mutants can also be activated. On the other hand, our experiments our inconclusive regarding the activation of T36A since this mutant does not bind Byr2(RBD) and does not promote mTORC2 pathway activation in cells stimulated by cAMP (Figs. 2 and 4*C*).

### RasC-CA mutants differentially affect mTORC2-dependent pPKBR1 in cells

**Figure 5:**
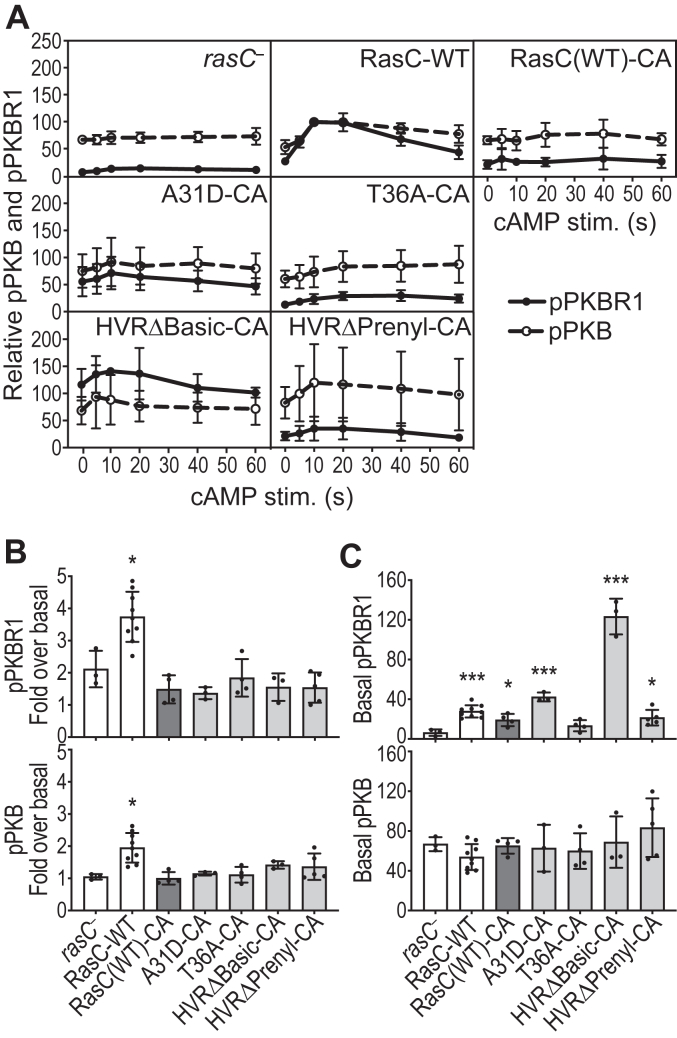
**The outcome of expressing wild-type and mutant RasC-CA forms on basal and cAMP-stimulated pPKB and pPKBR1 in cells.** Basal and cAMP-stimulated pPKB and pPKBR1 were assessed in *rasC* null cells expressing the CA forms of the A31D, T36A, HVRΔBasic, and HVRΔPrenyl mutants, compared to RasC-WT and RasC(WT)-CA expressing cells (Fig. S4). pPKB and pPKBR1 were detected by immunoblot and quantified by densitometry as described in the Experimental procedures section. Data on graphs represent measured pPKB and pPKBR1 ± S.D of at least three independent experiments for each strain. *A*, kinetics of cAMP-induced pPKB and pPKBR1 levels expressed relative to the peak activity of RasC-WT (10 s). *B*, pPKB and pPKBR1 levels after 10 s cAMP stimulation of each strain expressed as fold over corresponding basal levels. *C*, comparison of the pPKB and pPKBR1 basal levels. Data in (*B* and *C*) were analyzed using unpaired two-tailed t-tests. Differences between the pPKB and pPKBR1 levels in *rasC* null cells and the other strains: ∗*p* < 0.05; ∗∗*p* < 0.005; ∗∗∗*p* < 0.0005.

To help differentiate between impaired cAMP-stimulated, GEF-mediated activation of the A31D, T36A, HVRΔBasic and HVRΔPrenyl mutants and an inability to activate mTORC2, we then assessed pPKB and pPKBR1 in *rasC* null cells expressing the CA form of these mutants. Of note, whereas we observed that the cAMP stimulation of RasC-WT expressing cells induced a 2-fold increase in pPKB and pPKBR1, RasC(WT)-CA-expressing cells completely failed to promote pPKB and pPKBR1 in response to cAMP stimulation. This observation then suggested that expressing constitutively active RasC proteins leads to mTORC2 pathway desensitization in cells (Figs. 5, *A* and *B*, S4). It was then not surprising to observe that the RasC-CA mutants also didn’t promote cAMP-stimulated pPKB and pPKBR1. However, we observed interesting effects of expressing the different RasC-CA constructs on the basal levels of pPKB and pPKBR1 (Fig. 5*C*). First, we found that the expression of RasC-WT and RasC(WT)-CA significantly increased pPKBR1 (and not pPKB) basal levels compared to those in *rasC* null cells, with a 1.5 times lower effect of RasC(WT)-CA compared to RasC-WT. Together, these observations suggest that: (1) expression of RasC-WT increases the stochastic activation of the mTORC2 pathway; (2) expression of RasC(WT)-CA activates the mTORC2 pathway but that this is probably limited by desensitization and/or downregulation; and (3) that RasC promotes mTORC2 activation at the membrane, leading to pPKBR1 (membrane anchored) and not pPKB (soluble) in the absence of cAMP stimulation. Indeed, without stimulating the cells, the RasG-PI3K pathway is not activated and consequently, PKB is not recruited to the plasma membrane. Hence, these observations support a model where RasC activates mTORC2 at the plasma membrane.

Interestingly, T36A-CA-expressing cells displayed pPKBR1 basal levels comparable to those in *rasC* null cells (no increase in pPKBR1); whereas A31D-CA, HVRΔBasic-CA, and HVRΔPrenyl-CA, displayed significantly elevated pPKBR1 basal levels, which were surprisingly high for HVRΔBasic-CA [6.5 times higher than RasC(WT)-CA] (Fig. 5*C*). The observation that expression of the T36A-CA mutant fails to induce an increase in basal pPKBR1 levels suggests that the T36 residue is important for mTORC2 pathway activation. However, because our result with RasC(WT)-CA suggests that the mTORC2 pathway becomes desensitized and/or downregulated by the expression of constitutively active RasC proteins, it is difficult to draw a conclusion from this experiment. To address this issue, we turned to an *in vitro* assay, which was previously described by Cai *et al.* (24).

### *In vitro* RasC-mTORC2-pPKB/pPKBR1 assay reveals RasC key determinants include SWI T36 residue and HVR

**Figure 6:**
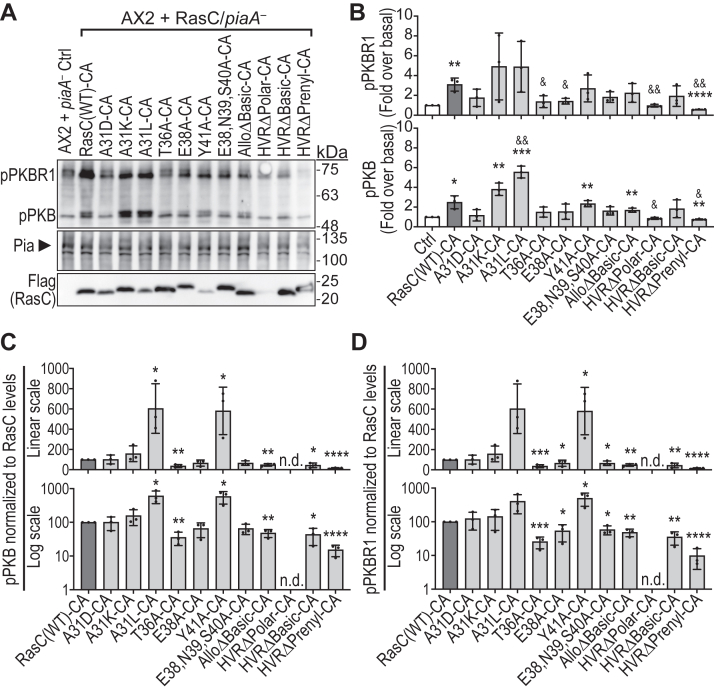
**Impact of the mutations on RasC-CA-stimulated and mTORC2-dependent pPKB and pPKBR1 *in vitro*.** Wild-type background RasC-CA [RasC(WT)-CA] or the indicated RasC mutants in their CA forms were individually expressed in *piaA* null cells (*piaA*^*-*^), mixed with wild-type AX2 cells, and then lysed mechanically as described in the Experimental procedures section. Non-transformed *piaA* null cells were used as negative control (Ctrl). pPKB and pPKBR1, as well as Pia (reflecting mTORC2 expression in AX2 cells) and the Flag-tagged RasC constructs were detected by immunoblot as described in the Experimental procedures section. *A*, immunoblots representative of three independent experiments. *B*, pPKB and pPKBR1 levels were quantified by densitometry, divided by Pia expression levels, and expressed as fold over the basal levels obtained with the negative control condition. Data were analyzed using unpaired two-tailed t-tests. Differences between pPKB and pPKBR1 levels in the control condition and reactions with a RasC construct: ∗*p* < 0.05; ∗∗*p* < 0.005; ∗∗∗*p* < 0.0005; ∗∗∗∗*p* < 0.0001. Differences between pPKB and pPKBR1 levels stimulated by RasC(WT)-CA and those stimulated by the RasC mutants: &*p* < 0.05; &&*p* < 0.005. *C* and *D*, pPKB and pPKBR1 levels normalized for Pia expression were corrected for RasC-CA construct expression levels. The data were plotted on both linear and log scale y axes to better visualize their range. Data were analyzed using unpaired two-tailed t-tests. Differences between pPKB and pPKBR1 levels stimulated by RasC(WT)-CA and those stimulated by the RasC mutants: ∗*p* < 0.05; ∗∗*p* < 0.005; ∗∗∗∗*p* < 0.0001. n.d., not determined.

To assess the RasC-mediated mTORC2 pathway activation *in vitro*, we expressed the CA form of wild-type RasC and each mutant in cells lacking the mTORC2 essential component Pia (*piaA* null; no mTORC2) and lysed them mechanically in the presence of wild-type AX2 cells (providing mTORC2 to the reaction), using non-transformed *piaA* null cells as negative control. We then measured the mTORC2-mediated pPKB and pPKBR1 in each reaction (Fig. 6*A*). Of note, we observed greater variations in the measured pPKBR1 than pPKB in this assay and although some of the significant differences measured for pPKB are non-statistically significant for pPKBR1, the overall effects on both kinases’ phosphorylation were similar.

When analyzing pPKB and pPKBR1 normalized to the detected Pia expression levels to account for the amount of mTORC2 in each reaction, we measured 2 to 4 fold significant stimulation of pPKB and pPKBR1 by RasC(WT)-CA over the control, indicative of RasC-dependent mTORC2 activation (Fig. 6*B*). Similarly, we detected considerable pPKB and pPKBR1 stimulations by A31K-CA, A31L-CA, Y41A-CA, and AlloΔBasic-CA mutants. By contrast, we observed significantly reduced pPKB and pPKBR1 with HVRΔPrenyl-CA compared to RasC(WT)-CA. To then also consider the differences in the amount of RasC construct in each reaction, we further normalized the quantified pPKB and pPKBR1 by the measured Flag-RasC levels. Note that this normalization was not possible for HVRΔPolar-CA due to this construct’s very low expression levels and, consequently, this experiment is inconclusive for this mutant. However, since HVRΔPolar promotes significant activation of the mTORC2 pathway in cells stimulated by cAMP (Fig. 2), we conclude that the polar residues Q174, N175, E176, and E177 in the HVR are not crucial for mTORC2 activation.

For the A31 mutants, our analysis showed that A31D-CA and A31K-CA stimulate mTORC2-dependent pPKB and pPKBR1 as efficiently as RasC(WT)-CA, and that A31L-CA is even more efficient, stimulating ∼5 times more pPKB and pPKBR1 (Fig. 6, *C* and *D*). The increased activity of A31L-CA towards mTORC2 *in vitro* is intriguing but consistent with our observations that this mutant also displays elevated activity and mediates strong mTORC2 pathway activation in cells (Figs. 2 and 4). Nonetheless, our results with these mutants indicate that residue A31 is not involved in selectively promoting mTORC2 activation in cells, but that its role in determining RasC’s signaling specificity lies at the level of its selective activation by the Aimless RasGEF. Indeed, when A31 is mutated into an aspartate as found at this position in RasG, which is not activated by Aimless, the RasC-A31D mutant does not undergo cAMP-stimulated activation (Fig. 4), while the CA form of this mutant effectively activates the mTORC2 pathway (Figs. 5*C* and 6*C*). Interestingly, previous studies of the human RasGEF SOS and its interaction with H-Ras showed that while the interaction core involves Ras’s SWII region, SOS also interacts with SWI (48). Furthermore, SWI, which normally interacts with the nucleotide, was found to be completely displaced from the nucleotide binding pocket upon binding of SOS. Therefore, considering our results, what is known about the RasGEF-Ras interaction, and previous studies on the role of residue A31 in RasC’s signaling specificity, we propose that: (1) A31 is important for Aimless binding and its activation of RasC; (2) that the presence of an aspartate at this position prevents its interaction with Aimless; and (3) that mutations of this residue into leucine, and possibly lysine, may destabilize SWI’s interaction with the nucleotide binding site and thereby facilitate GDP release and Ras activation.

For the effector domain mutants, we observed that the mTORC2-dependent pPKB and pPKBR1 levels stimulated by T36A-CA were reduced by ∼70% compared to RasC(WT)-CA, thereby further supporting a role for this residues in mediating mTORC2 activation (Fig. 6*C*). On the other hand, the E38A-CA and E38,N38,S40A-CA mutants showed a much smaller effect, with only significantly reduced pPKBR1 by ∼40% compared to RasC(WT)-CA. This result is also consistent with our observation that the E38A and E38,N39,S40A mutants partially rescue mTORC2 pathway activation in *rasC* null cells (Fig. 2). Of note, these two mutants behaved in extremely similar ways in all of our experiments. Thus, we conclude that the effects observed in the triple E38,N39,S40A mutant are likely due to the E38A mutation alone, which plays a minor role in promoting mTORC2 pathway activation, and that N39 and S40 are not involved in mediating RasC function in cells. However, it is interesting that, while these mutants partly rescue mTORC2 pathway activity, they completely fail to rescue the aggregation defects of *rasC* null cells. Therefore, these observations support a role for RasC in *Dictyostelium* aggregation that is partly independent of mTORC2. Interestingly, we observed that the Y41A-CA mutant strongly stimulated pPKB and pPKBR1 in our *in vitro* assay, to levels ∼5 times higher than those stimulated by RasC(WT)-CA, an effect similar to that of the A31L-CA mutant (Fig. 6*C*). Therefore, this observation not only suggests that Y41 is not important for mediating mTORC2 activation but that its mutation potentially increases the activity of RasC.

Our results with RasC effector domain mutants T36A and E38A (as well as E38,N39,S40A) that suggest a major role for T36 and a minor one for E38 are consistent with previous findings with human Ras. Indeed, Kovalski *et al.* showed that T35 (T36 in RasC) plays an important role in H-Ras’s interaction with mTOR and in promoting mTORC2 signaling in cells, and that E37 (E38 in RasC) is not involved (4). The same study also suggested a role for residue Y40 (Y41 in RasC) of H-Ras for its interaction with and activation of mTORC2, but we didn’t observe this effect for *Dictyostelium* RasC. Interestingly, the study by Kovalski *et al.* also suggests that two Ras molecules bind mTORC2 through their effector domains and that both are necessary for mTORC2 activation in cells: one Ras binds the mTORC2 unique component SIN1 through its atypical RBD and another Ras binds mTOR’s kinase domain (4). However, Ras binding to SIN1 for mTORC2 regulation is debated, as a more recent study showed that this interaction is dispensable for mTORC2 function in cells (6), and we found that RasC does not bind RIP3, the *Dictyostelium* ortholog of SIN1 (15). Instead, we previously showed that RIP3 binds another small GTPase, Rap1, and that this interaction is conserved for human Rap1 and SIN1 (15). Consequently, we previously proposed a context-dependent model of Ras-regulated mTORC2 activity in which one signal is necessary to localize mTORC2 at the plasma membrane through SIN1, whether it involves its binding to a small GTPase with its RBD or PI(3,4,5)P_3_ through its PH domain, and Ras binding to mTOR (49). Here, our observations suggest a particularly important role for residue T36 in RasC’s effector domain to promote mTORC2 activation and, since we previously showed that RasC does not interact with SIN1/RIP3, it is possible that this residue could be implicated in an interaction of RasC with mTOR. However, further studies are needed to confirm this hypothesis.

In our *in vitro* assay, the allosteric domain mutant AlloΔBasic-CA showed a ∼50% reduced pPKB and pPKBR1 stimulation compared to RasC(WT)-CA (Fig. 6*C*). This observation is consistent with the ability of this mutant to only partially rescue the aggregation and mTORC2 pathway activation in *rasC* null cells (Figs. 1*D* and 2). This result then supports a role for residue(s) K138, R164, and/or K167 for optimal RasC-mediated activation of mTORC2 but also indicates that these residues are not essential. Interestingly, before completion of our study, Senoo *et al.* reported the interaction of RasC with Rho GTPase RacE oligomers involving residue R164 of RasC, and that this RasC:RacE complex is necessary for mTORC2 activation, with evidence that this mechanism is conserved in human cells (40, 41). Hence, we propose that the partial loss of RasC function in the AlloΔBasic mutant is likely the result of a reduced binding to RacE.

Finally, we observed that the HVR mutations in HVRΔBasic-CA and HVRΔPrenyl-CA strongly impaired the ability of RasC to stimulate the mTORC2-dependent pPKB and pPKBR1 *in vitro*, with levels reduced by ∼60% and ∼90%, respectively (Fig. 6*C*). Interestingly, these effects strongly correlate with the impaired membrane association of these mutants (Fig. 3). Since we also found that the HVRΔBasic and HVRΔPrenyl RasC mutants do not undergo cAMP-induced activation (Fig. 4), these results indicate that RasC membrane anchoring is crucial for both its own activation in response to cAMP stimulation and for the downstream RasC-mediated activation of mTORC2. Whether the importance of RasC membrane anchoring on the RasC-mTORC2 pathway is due to localizing RasC to the plasma membrane, or that it is necessary for RasC to adopt a conformation favoring its interaction with the Aimless RasGEF and mTORC2, or both, remains to be determined.

## Conclusion

**Figure 7:**
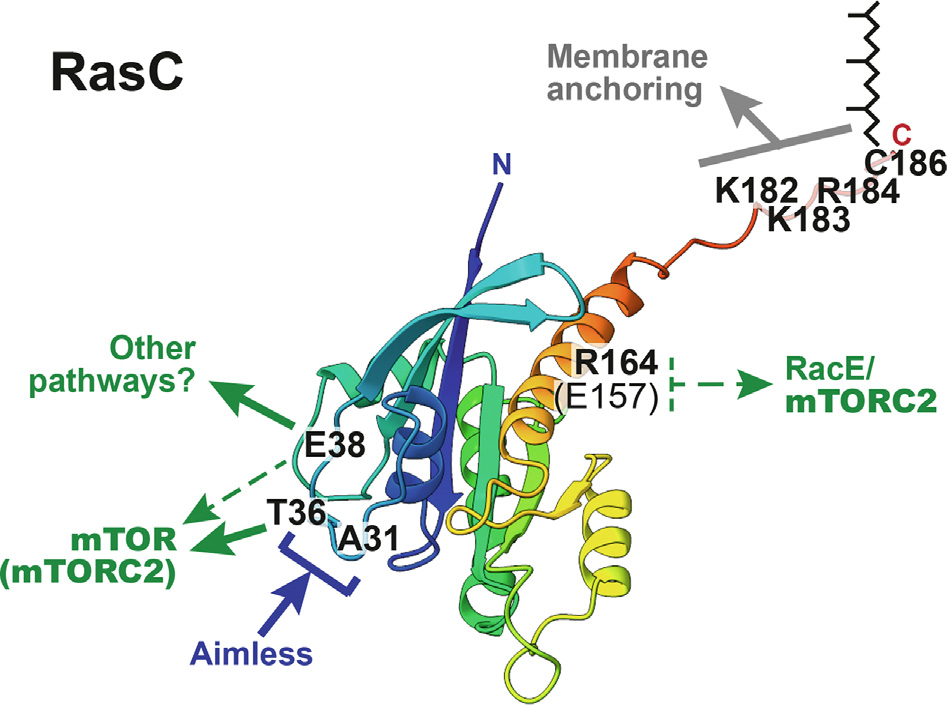
**Key roles for specific SWI and allosteric domain residues, as well as of the HVR in RasC regulation and signaling.** A predicted structure of RasC is shown as a rainbow ribbon, generated using the Robetta server and UCSF ChimeraX (61, 62). Since Ras protein HVRs are unstructured, we chose to display an extended RasC HVR in this model. In this RasC structure prediction, the SWI loop is colored *cyan*, the C-terminal allosteric domain includes two α helices (α4, *yellow* and α5, *orange*) linked by a short β strand and loop, and the HVR is *colored red*. The residues that we found to be implicated in RasC regulation and function are indicated and bolded. First, we found that membrane anchoring, mediated by the C-terminal HVR (particularly implicating residues K182, K183, and R184, and farnesylation site C186), is essential for both the cAMP-induced activation of RasC, which is specifically promoted by the Aimless RasGEF as part of the Sca1 complex, and for the RasC’s downstream activation of mTORC2. Our results also revealed an important role for SWI residue A31 in the Aimless RasGEF-mediated activation of RasC, which appears to regulate the Aimless recognition of RasC. On the other hand, we identified residues in the effector domain of SWI, T36 and E38, as well as in the allosteric domain of RasC that mediate its function in cells. Our results suggest that T36 is important for mTORC2 activation, as was shown for human Ras (4), whereas E38 plays a minor role in mTORC2 activation but its importance for *Dictyostelium* aggregation suggests the involvement of other RasC-dependent pathways. Allosteric domain residue R164 likely regulates mTORC2 activation by promoting RasC’s interaction with RacE, along with residue E157 that was identified by Senoo *et al.* (41).

Our study reveals key determinants of RasC’s specific regulation and activation of mTORC2 in *Dictyostelium*. First, altogether, our data revealed that RasC’s membrane anchoring, mediated by the combination of electrostatic interactions from residues K182, K183, and R184 and farnesylation at residue C186, is necessary for both its own activation and the downstream regulation of mTORC2. Second, our data suggest that after the cAMP-induced recruitment of the Sca1 complex to the plasma membrane (25), the selective recognition of plasma membrane-anchored RasC by the Aimless RasGEF likely involves SWI interactions regulated by residue A31 (Fig. 7). Importantly, RasG displays an aspartate at position 31 and our data suggest that this prevents RasC activation, providing an explanation for the selective activation of RasC by Aimless. Finally, our results also revealed determinants of the RasC-mediated mTORC2 activation. Considering our findings together with those from previous studies of *Dictyostelium* and mammalian Ras and mTORC2 that were published while we were conducting this work, we propose the following: Aimless-activated RasC interacts with RacE through its allosteric domain, involving residue R164 (as well as E157 as found by Senoo *et al.*), and with mTOR through its effector domain, involving residue T36 and to a lesser extent E38, to promote efficient mTORC2 activation at the plasma membrane.

We expect that there are other determinants of RasC-mTORC2 signaling in addition to those that we and others have identified so far. For example, it is likely that other SWI residues are important for mediating mTORC2 activation, such as P35 and I37 which correspond to residues P37 and I39 in Rheb previously shown to promote its binding to mTOR (50, 51), as well as other residues in the allosteric domain such as R149, D153, and Y157 found to be involved in the interaction of human K-Ras with the cysteine-rich domain of Raf1 (52). Therefore, additional studies are necessary to obtain a complete picture of the mechanism through which Ras promotes mTORC2 activation. Nonetheless, our studies in *Dictyostelium* significantly contribute to our understanding of the selectivity in eukaryotic Ras protein signaling and we expect that our findings will help understand the role and regulation of Ras function in human cells.

## Experimental procedures

### DNA constructs

Wild-type Flag-RasC cloned in the *Dictyostelium* extra-chromosomal expression vector pDM304 was described previously (53). Flag-RasC was subcloned in the pBluescript SK- vector for use in site-directed mutagenesis to generate the 11 RasC mutants indicated in Figure 1*B* as well as the constitutively active (CA) RasC-Q62L mutant. Except for RasC-AlloΔBasic, which was performed in 2 sequential steps, all other mutants were generated in one step using the following oligonucleotides:

A31D forward, CTATTCAATTAACACAAAATCAATTCATTGATGAATATGATCCAACTATAGAA AACTCT.

A31D reverse, AGAGTTTTCTATAGTTGGATCATATTCATCAATGAATTGATTTTGTGTTAATTG AATAG.

A31K forward, CACTTACTATTCAATTAACACAAAATCAATTCATTAAGGAATATGATCCAAC TATAGAAAACTCTTATCGTAA.

A31K reverse, TTACGATAAGAGTTTTCTATAGTTGGATCATATTCCTTAATGAATTGATTTTGT GTTAATTGAATAGTAAGTG.

A31L forward, CTTACTATTCAATTAACACAAAATCAATTCATTCTAGAATATGATCCAACTAT AGAAAACTCTTATCGT.

A31L reverse, ACGATAAGAGTTTTCTATAGTTGGATCATATTCTAGAATGAATTGATTTTGTGT TAATTGAATAGTAAG.

T36A forward, CAATTCATTGCTGAATATGATCCAGCTATAGAAAACTCTTATCGTAAAC.

T36A reverse, GTTTACGATAAGAGTTTTCTATAGCTGGATCATATTCAGCAATGAATTG.

E38A forward, ATTGCTGAATATGATCCAACTATAGCAAACTCTTATCGTAAACAAGTAAAC.

E38A reverse, GTTTACTTGTTTACGATAAGAGTTTGCTATAGTTGGATCATATTCAGCAAT.

Y41A forward, CATTGCTGAATATGATCCAACTATAGAAAACTCTGCTCGTAAACAAGTAAAC ATTG.

Y41A reverse, CAATGTTTACTTGTTTACGAGCAGAGTTTTCTATAGTTGGATCATATTCAGCA ATG.

E38A,N39A,S40A forward, GAATATGATCCAACTATAGCAGCCGCTTATCGTAAACAAGTAAA CATTG.

E38A,N39A,S40A reverse, CAATGTTTACTTGTTTACGATAAGCGGCTGCTATAGTTGGATCAT ATTC.

K138 (AlloΔBasic-step 1) forward, CTAAAAATGGTGCACCAAAACTTGCTGCTAACTCCTTAC CCTCCATTG.

K138 (AlloΔBasic-step 1) reverse, CAATGGAGGGTAAGGAGTTAGCAGCAAGTTTTGGTGCAC CATTTTTAG.

R164A,R167A (AlloΔBasic-step 2) forward, CGTTTTGTGGATTTTGATTCCATCTTGCAATTTCT GCAACGAGGGTAAAGAAGGCCTCTTCAAC.

R164A,R167A (AlloΔBasic-step 2) reverse, GTTGAAGAGGCCTTCTTTACCCTCGTTGCAGAAA TTGCAAGATGGAATCAAAATCCACAAAACG.

Q174A, N175A, E176A,E177A (HVRΔPolar) forward, ACATCCCCTTTTCTTTGGTGGGAGCATC GCTGCGGCTGCTGGATTTTGATTCCATCTTTTAATTTCTCTAAC.

Q174A, N175A, E176A,E177A (HVRΔPolar) reverse, GTTAGAGAAATTAAAAGATGGAATCAA AATCCAGCAGCCGCAGCGATGCTCCCACCAAAGAAAAGGGGATGT.

K182A,K183A,R184A (HVRΔBasic) forward, CTCTAGAACTAGTTTACAATATAATACATCCCG CTGCCGCTGGTGGGAGCATCTCTTCGTTTTGTGGATTTTGATT.

K182A,K183A,R184A (HVRΔBasic) reverse, AATCAAAATCCACAAAACGAAGAGATGCTCCC ACCAGCGGCAGCGGGATGTATTATATTGTAAACTAGTTCTAGAG.

C186A (HVRΔPrenyl) forward, CTAGAACTAGTTTACAATATAATAGCTCCCCTTTTCTTTGGTG GGAGCA.

C186A (HVRΔPrenyl) reverse, TGCTCCCACCAAAGAAAAGGGGAGCTATTATATTGTAAACTA GTTCTAG.

Q62L (RasC-CA) forward, ATTTTAGATACAGCCGGTCTAGAAGAGTATAGCGCTATG.

Q62L (RasC-CA) reverse, CATAGCGCTATACTCTTCTAGACCGGCTGTATCTAAAAT.

Successful RasC mutation was confirmed by sequencing. Each RasC construct was then transferred by PCR to pDM304, which was obtained from the Dicty Stock Center and described previously (54, 55, 56), and the final constructs were verified again by sequencing. The oligonucleotides used to transfer the Flag-RasC constructs from pBluescript SK- to pDM304 are the following, with different reverse oligonucleotides used for the HVRΔBasic and HVRΔPrenyl:

Flag-RasC pDM304 transfer forward, AAGTGAGATCTAAAAAATGGACTACAAAGACGATGAC.

Flag-RasC pDM304 transfer reverse, AAGTGACTAGTTTACAATATAATACATCCCCTTTTCTTTG GTGGG.

HVRΔBasic pDM304 transfer reverse, AAGTGACTAGTTTACAATATAATACATCCCGCTGCC.

HVRΔPrenyl pDM304 transfer reverse, AAGTGACTAGTTTACAATATAATACATCCCGCTGCC.

The additional mutation of Q62L on the 11 RasC mutant constructs was then generated using the GoldenBraid system (57). For this, each Flag-RasC mutant was first transferred from pDM304 to the entry plasmid pUPD2 using the following oligonucleotides:

Flag-RasC pUPD2 transfer forward, GCGCCGTCTCACTCGAATGGACTACAAAGACGATGACG ACAAGAATTCC.

Flag-RasC pUPD2 transfer reverse, GCGCCGTCTCACTCGAAGCTTACAATATAATACATCCCCT TTTCTTTGGTGGG.

HVRΔBasic pUPD2 transfer reverse, GCGCCGTCTCACTCGAAGCTTACAATATAATACATCCCG CTGCCGCTGGTGGGAGCATCTCTTCGTTTTGTGG.

HVRΔPrenyl pUPD2 transfer reverse, GCGCCGTCTCACTCGAAGCTTACAATATAATAGCTCCC CTTTTCTTTGGTGGGAGCATCTCTTCG.

The Q62L mutation was then added to each Flag-RasC construct by site-directed mutagenesis using the Q62L (RasC-CA) oligonucleotides described above. The final expression plasmids were then assembled with the actin 15 promotor and actin 8 terminator (same ones as in the pDM304 vector), the FTU vector for *Dictyostelium* extrachromosomal maintenance and replication, and the pDGBΩ1 vector with neomycin resistance cassette, following the protocol previously described by Kundert *et al.* (57).

### Cell culture

The *Dictyostelium* strains used are AX2 (wild-type), *rasC* null cells, and *piaA* null cells provided by Peter Devreotes and described elsewhere (24, 58). The cells were grown attached to the substrate in axenic HL5 medium including glucose (ForMedium) at 20 to 22 °C and transformants were generated by electroporation. Transformed cells were selected in 20 μg/ml Geneticin (GIBCO; Thermo Fisher Scientific) and protein expression was confirmed by immunoblot. For experiments, cells were amplified in shaking cultures for 24 to 48 h to 2 to 4 × 10^6^ cells/ml. To obtain cAMP-responsive differentiated cells, these were developed by pulsing with 30 nM cAMP (cAMP sodium salt monohydrate; Millipore Sigma) every 6 min for 5.5 h at 5 × 10^6^ cells/ml in 12 mM Na/K phosphate buffer (2.4 mM Na_2_HPO_4_, 9.6 mM KH_2_PO_4_, pH 6.1).

### Development

Log-phase growing cells were collected, washed with 12 mM Na/K phosphate buffer and resuspended at 20 × 10^6^ cell/ml. Cells were then plated on non-nutrient agar and allowed to develop for 48 h and imaged using a Motic digital microscope (model DM-143).

### Cellular mTORC2 pathway activation

To assess the cellular phosphorylation of PKB and PKBR1, 5 × 10^7^ of 5.5 h-developed cells were washed twice with 12 mM Na/K phosphate buffer, resuspended in 1 ml buffer, and incubated 30 min with shaking prior to stimulation with 1 μM cAMP. 90 μl samples were collected at 0, 5, 10, 20, 40, and 60 s and immediately mixed with 30 μl 4X Laemmli sample buffer (final: 50 mM Tris pH 6.8, 50 mM DTT, 1% bromophenol blue, 1% SDS, and 5% glycerol). The protein samples were resolved on 12% SDS-PAGE and transferred to nitrocellulose membranes. Phospho-PKB (pPKB) and -PKBR1 (pPKBR1) were detected by immunoblot using a custom-made Rabbit polyclonal antibody directed against the phosphorylated hydrophobic motif of PKBR1 [KDTSFEGF(pT)YVADSC] generated by BIOMATIK. Immunoblots of the expressed Flag-RasC constructs were performed using anti-Flag M2 mouse monoclonal antibody from Millipore Sigma and immunoblot of actin, used as loading control, was performed using anti-beta-actin (C4) monoclonal Ab from Santa Cruz Biotechnology. The immunoblots were quantified by densitometry using ImageJ/Fiji (59, 60). Levels of pPKB and pPKBR1 in each sample were then normalized by dividing them by the corresponding actin levels, and then expressed as percentage of the 10 s peak stimulation in control cells expressing the wild-type Flag-RasC from each experiment.

### Membrane-cytosol fractionation

5 × 10^8^ of 5.5 h-developed cells were washed twice with cold 12 mM Na/K phosphate buffer, resuspended in 3 ml cold membrane separation lysis buffer (20 mM Tris pH 7.5, 5 mM EDTA, 5 mM DTT, 4 μg/ml aprotinin, and 4 μg/ml leupeptin), and incubated on ice for 10 min. The cells were then mechanically lysed on ice using a Polytron (Kinematica GmbH p110/35 series 11,881) on setting 6, homogenizing twice for 20 s. Lysates were centrifuged at 500*g* for 5 min at 4 °C and the supernatants were transferred to new tubes. 180 μl samples of the cleared lysates were collected and mixed with 60 μl 4X Laemmli sample buffer (total cell lysates). Membranes from 2 ml of the remaining cell lysates were then pelleted by centrifugation at 100,000*g* for 40 min at 4 °C. The supernatants were transferred to new tubes and 180 μl samples were mixed with 60 μl 4X Laemmli sample buffer (cytosol fractions). The membrane pellets were resuspended directly in 500 μl denaturation buffer (100 mM Tris pH 6.8, 100 mM DTT, 4% SDS, and 10% glycerol) using insulin syringes to break up the pellet completely, and 50 μl of the membrane samples were then collected and diluted 1:4 with 12 mM Na/K phosphate buffer and 4X Laemmli sample buffer to allow for direct comparison with proteins in the cytosol fractions and total cell lysate samples. 20 μl protein samples from the total cell lysates, membrane fractions, and cytosol fractions, were then resolved on 12% SDS-PAGE and transferred to nitrocellulose membranes. Flag-RasC in the different fractions was detected by immunoblot using anti-Flag M2 antibody and the immunoblot of actin in total lysates was used as loading control. The immunoblots were quantified by densitometry using ImageJ/Fiji (59, 60). Measurements of Flag-RasC for membrane and cytosol fractions for each condition were summed to generate total Flag-RasC levels, and the relative proportion of Flag-RasC detected in each fraction was calculated as a percentage of total Flag-RasC for each condition.

### Active RasC pull-down

To measure the cAMP-induced activation of Flag-RasC in cells, 5 × 10^8^ of 5.5 h-developed cells were washed twice with 12 mM Na/K phosphate buffer and resuspended in 5 ml buffer. The cell suspension was then incubated for 30 min with shaking before stimulation with 1 μM cAMP. 500 μl samples were collected before and at 5, 10, 20, 40, and 60 s after stimulation and mixed with 500 μl of cold 2X Ras lysis buffer (100 mM Tris pH 7.5, 300 mM NaCl_2_, 50 mM MgCl_2_, 20% glycerol, and 1% NP-40 supplemented with 4 μg/ml aprotinin and leupeptin) on ice. After a 10 min incubation on ice with frequent vortexing, samples were cleared by centrifugation at 20,000*g* for 10 min at 4 °C and the supernatants were transferred to new tubes. 60 μl aliquots were collected and mixed with 20 μl 4X Laemmli sample buffer for the analysis of Ras expression in total cell lysates, and the remainder of the lysates were incubated with 2 mg/ml BSA and 10 μg of previously prepared agarose beads coated with the Ras binding domain (RBD) of the yeast Ras effector Byr2 fused to GST [Byr2(RBD)], as described previously (47), for 1 h at 4 °C with agitation. The beads were then washed three times with 1 ml cold Ras lysis buffer and eluted in 30 μl of 2X Laemmli sample buffer.

To assess the ability of the RasC mutants to bind Byr2(RBD) independently of their ability to be activated by cAMP stimulation, 5 × 10^7^ of log-phase growing cells expressing the Flag-RasC mutants harboring the additional constitutively active mutation Q62L were washed twice with cold 12 mM Na/K phosphate buffer and resuspended in 500 μl buffer. The cells were lysed by mixing with 500 μl 2X Ras lysis buffer and incubated on ice for 10 min with frequent vortexing. The lysates were then cleared by centrifugation, total Ras samples were collected, lysates incubated with GST-Byr2(RBD) beads, and the pulled-down Ras proteins were eluted as described above.

Proteins from the pulled-down and total cell lysate samples were then resolved on 12% SDS-PAGE and transferred to nitrocellulose membranes. Flag-RasC was detected by immunoblot using anti-Flag M2 antibody and the immunoblots were quantified by densitometry using ImageJ/Fiji (59, 60). Flag-RasC detected in the pull-down samples for each condition was normalized to its corresponding total Flag-RasC and the activity level of the Flag-RasC mutants was expressed as a percentage of the 10 s peak activity of wild-type Flag-RasC.

### *In vitro* mTORC2 pathway activation assay

The ability of the RasC mutants to activate mTORC2 independently of their own GEF-mediated activation was assessed using an *in vitro* mTORC2-dependent PKB/PKBR1 phosphorylation reconstitution assay as described previously by Cai *et al.* (24). For this, each Flag-tagged RasC mutants containing the additional constitutively active Q62L mutation was expressed in *piaA* null cells (no functional mTORC2) and developed for 5.5 h, alongside wild-type AX2 cells. The cells were then incubated with 5 mM caffeine (Millipore Sigma, St Louis, MO, USA) for 20 min at 22 °C to bring all signaling activities due to autocrine cAMP stimulation to basal levels. The cells were then washed and resuspended in cold PM buffer (5 mM Na_2_HPO_4_, 5 mM KH_2_PO_4_, and 2 mM MgSO_4_) at 8 × 10^7^ cells/ml and kept on ice. 50 μl of AX2 cells were then mixed with 50 μl of RasC-CA wild-type or RasC mutant-CA expressing *piaA* null cells, or only *piaA* null cells as negative control. The combined cell suspensions were then mixed with 100 μl TM buffer (20 mM Tris pH 8.0 and 2 mM MgSO_4_) and 1 μl 60 mM ATP (300 μM final) and lysed mechanically by passing them through a 3 μm filter. Reactions were then incubated on ice for 10 min and stopped by the addition of 20 μl 6X Laemmli sample buffer. Immunoblots of pPKB and pPKBR1 and Flag-RasC constructs were performed and analyzed as described for the cellular mTORC2 pathway activation experiment. Immunoblots of Pia were performed using a custom-made Pia antibody previously described (53). The immunoblots were quantified by densitometry using ImageJ/Fiji (59, 60). To normalize the levels of pPKB and pPKBR1, we first divided by the corresponding levels of Pia and expressed them as fold over basal, and then further divided by the corresponding levels of RasC constructs expression and expressed the pPKB and pPKBR1 levels as percentage of the stimulation by the RasC-CA control from each experiment.

### Statistical analyses

All quantified data were graphed and analyzed in Prism using two-tailed unpaired t-tests for data sets with sample size = 3 or to make individual comparisons, and one-way ANOVA test for data sets with sample size > 3 followed by appropriate *post hoc* multiple comparison analyses when applicable. The *p* values or adjusted *p* values are indicated in the figure legends, and a *p* of 0.05 was considered the statistically significant threshold.

## Data availability

All data are contained within the main manuscript and supporting information.

## Supporting information

This article contains supporting information (63, 64).

## Conflict of interest

The authors declare that they have no conflicts of interest with the contents of this article.
