# Supplementary material for: Molecular determinants of Ras-mTORC2 signaling
Source: J Biol Chem. 2024 May 28;300(7):107423. doi: 10.1016/j.jbc.2024.107423 (PMC11255897; doi:10.1016/j.jbc.2024.107423)
Supplement: Supporting Information [file mmc1.pdf]

Supporting information for:

Molecular determinants of RasC-mTORC2 signaling in *Dictyostelium*

Stephen F. Smith[2,4], A.F.M. Tariqul Islam[1], Shoxruxxon Alimukhamedov[1,5], Ethan T. Weiss[1,6] and Pascale G. Charest[1,2,3]*

[1] Department of Molecular and Cellular Biology, University of Arizona, Tucson, AZ, USA
[2] Department of Chemistry and Biochemistry, University of Arizona, Tucson, AZ, USA
[3] University of Arizona Cancer Center, Tucson, AZ, USA
[4] Current address: Department of Pharmacology and Toxicology, University of Arizona, Tucson, AZ, USA
[5] Current address: Department of Neurology and Neurological Sciences, Stanford University School of Medicine, CA, USA
[6] Current address: Illumina, San Diego, CA, USA
* Correspondence: Pascale G. Charest (pcharest@arizona.edu)

List of material included:

Supporting Figure S1……page S-1
Supporting Figure S2……page S-2
Supporting Figure S3……page S-3
Supporting Figure S4……page S-4

References

(63) Sievers, F., Wilm, A., Dineen, D., Gibson, T. J., Karplus, K., Li, W., Lopez, R., McWilliam, H., Remmert, M., Söding, J., Thompson, J. D., and Higgins, D. G. (2011) Fast, scalable generation of high-quality protein multiple sequence alignments using Clustal Omega. Mol. Syst. Biol. 7, 539

(64) Waterhouse, A. M., Procter, J. B., Martin, D. M. A., Clamp, M., and Barton, G. J. (2009) Jalview Version 2—a multiple sequence alignment editor and analysis workbench. Bioinformatics. 25, 1189–1191

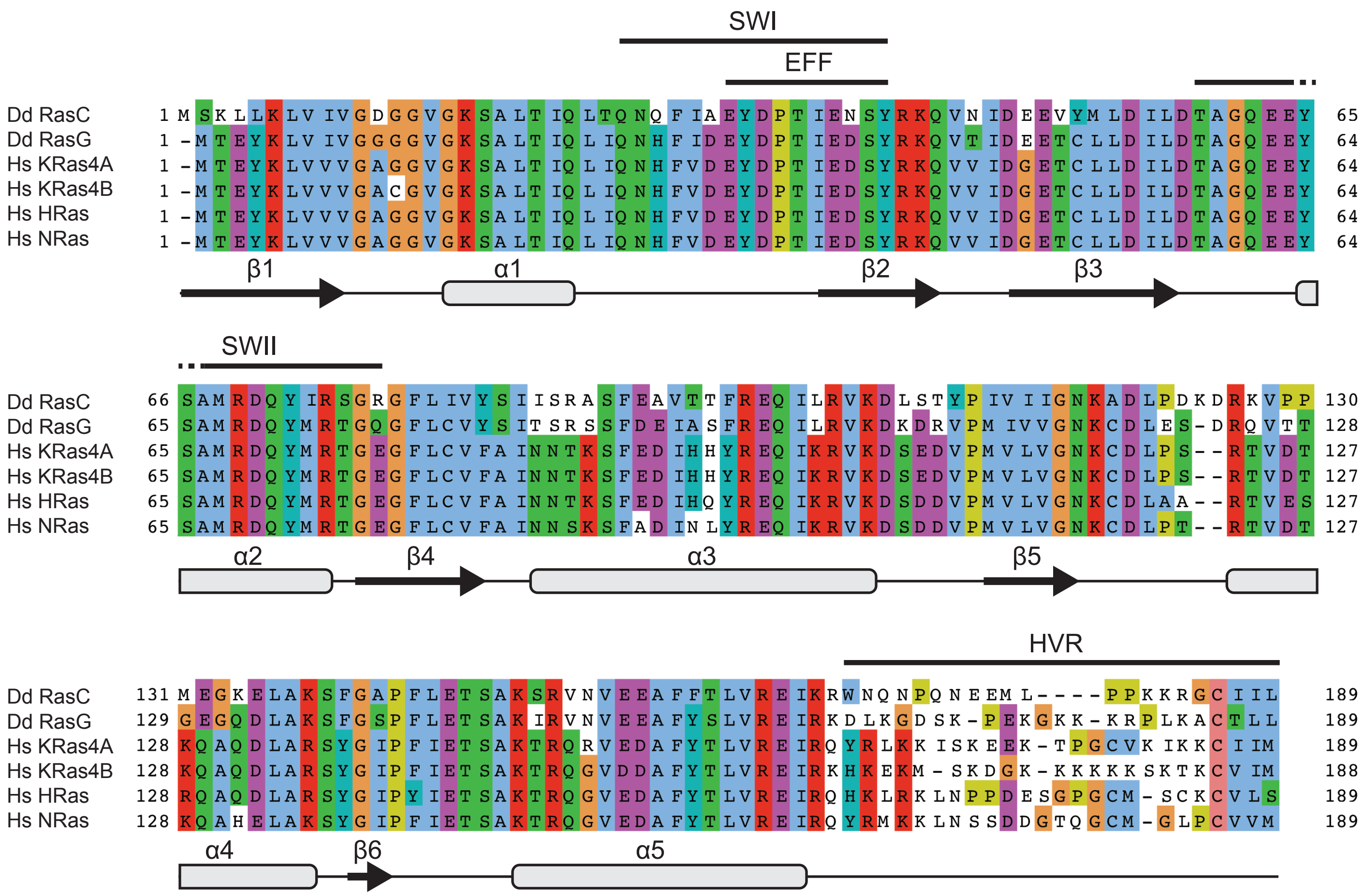

Supporting figure 1. Sequence comparison of *Dictyostelium* RasC with other Ras family GTPases. The sequence of *Dictyostelium discoideum* (Dd) RasC was aligned to that of Dd RasG and Homo sapiens (Hs) Ras proteins KRas4A, KRas4B, HRas, and NRas. Locations of the secondary protein structures beta sheets and alpha helices as well as the switch I (SWI; ~25-40) and II (SWII; ~58-77) domains, canonical effector binding domain (EFF; ~31-40) and hypervariable region (HVR; ~66-189) are indicated. The alignment was generated using Clustal Omega and JalView (62-63).

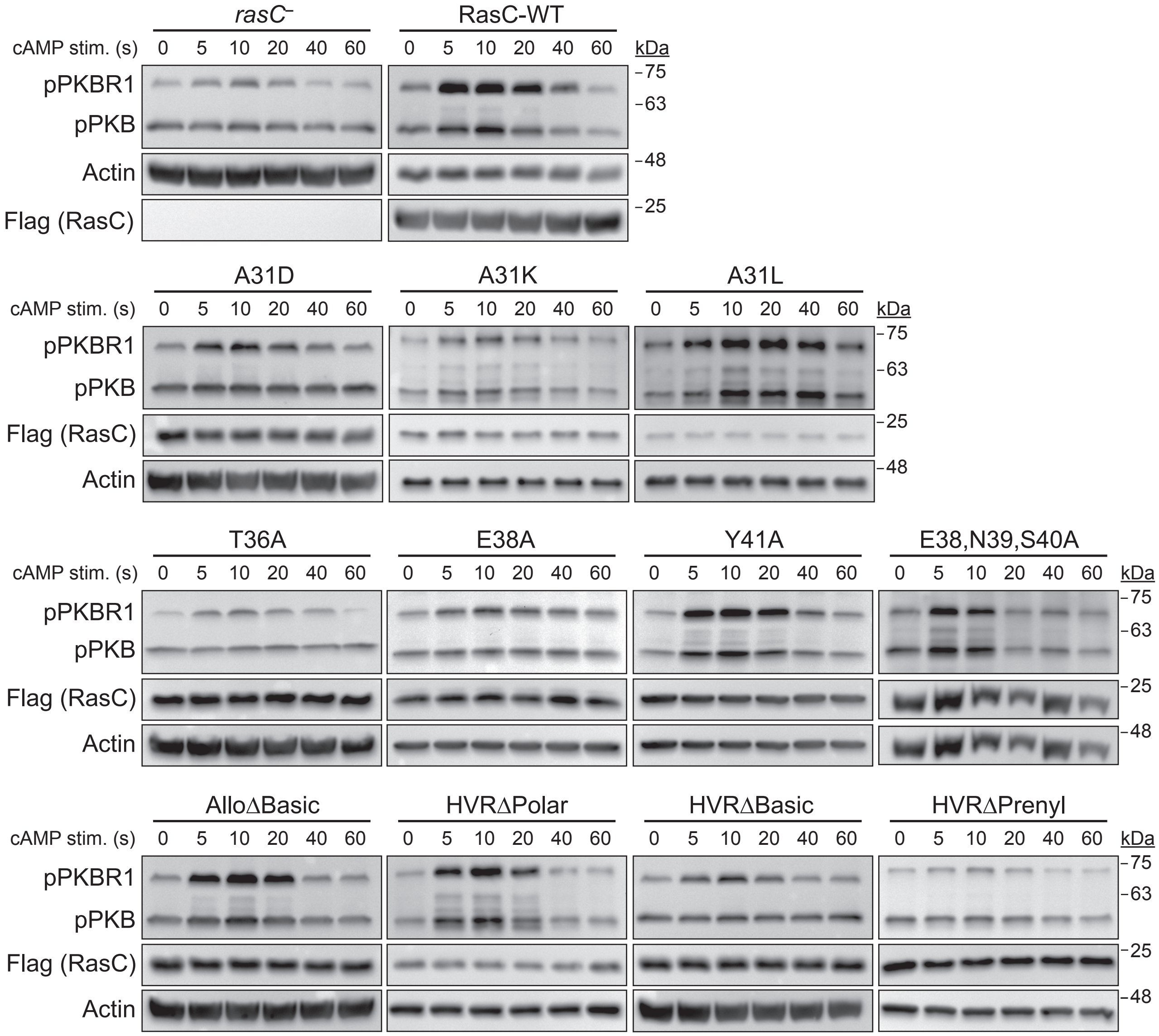

Supporting figure 2. Impact of the RasC mutations on mTORC2-mediated pPKB and pPKBR1 in cells. *rasC* null (*rasC*–) cells or *rasC* null cells expressing the indicated Flag-tagged RasC proteins were developed and stimulated with cAMP for the indicated time before cells were lysed and phosphorylation of PKB (pPKB) and PKBR1 (pPKBR1) were revealed by immunoblot as described in the Experimental procedures section. Flag (RasC) and actin immunoblots were used as expression and loading controls, respectively. Immunoblots shown are representative of at least 3 independent experiments for each strain, which were quantified in main Figure 2.

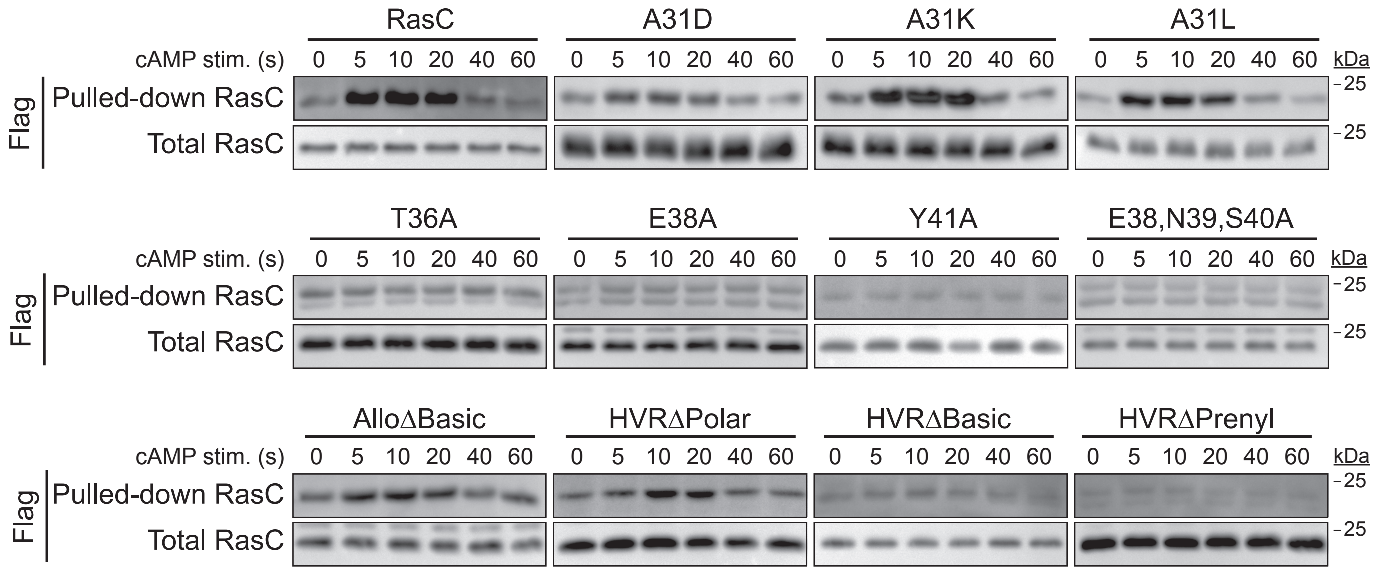

Supporting figure 3. Differential effects of the RasC mutations on their own cAMP-induced activation and binding to a canonical effector. *rasC* null (*rasC*⁻) cells expressing the different Flag-tagged RasC proteins were stimulated with cAMP for the indicated time before cells were lysed and RasC activity assessed in a pull-down assay using Byr2(RBD) as described in the Experimental procedures section. Pulled-down, active RasC and total RasC were revealed by Flag immunoblot. Immunoblots shown are representative of at least 3 independent experiments for each strain, which were quantified in main Figure 4.

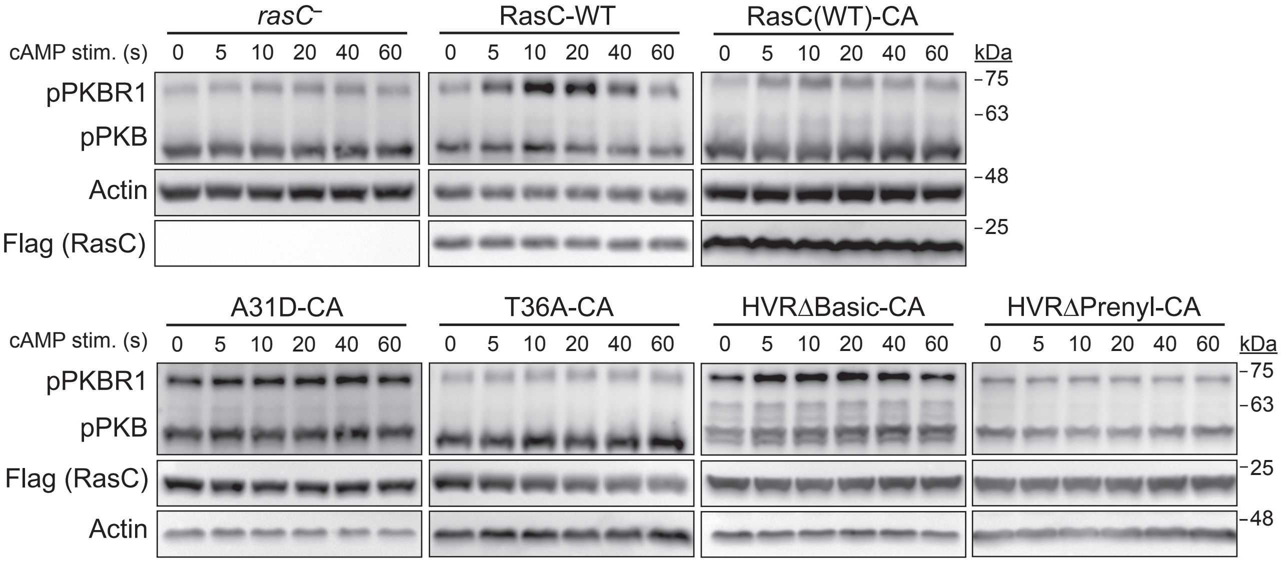

Supporting figure 4. The outcome of expressing wild-type and mutant RasC-CA forms on pPKB and pPKBR1 levels in cells. *rasC* null (*rasC*–) cells or *rasC* null cells expressing the indicated Flag-tagged RasC proteins, with or withour the additional constitutively active (CA) mutation Q62L, were developed and stimulated with cAMP for the indicated time before cells were lysed and phosphorylation of PKB (pPKB) and PKBR1 (pPKBR1) were revealed by immunoblot as described in the Experimental procedures section. Flag (RasC) and actin immunoblots were used as expression and loading controls, respectively. Immunoblots shown are representative of at least 3 indpendent experiments for each strain, which were quantified in main Figure 5.
